# A cross-country comparison of tobacco consumption among youths from selected South-Asian countries

**DOI:** 10.1186/1471-2458-13-379

**Published:** 2013-04-23

**Authors:** Mohammad Alamgir Kabir, Kim-Leng Goh, Mobarak Hossain Khan

**Affiliations:** 1 Department of Applied Statistics, Faculty of Economics and Administration, University of Malaya, Kuala Lumpur, 50603, Malaysia; 2 Department of Statistics, Jahangirnagar University, Savar, Dhaka, 1342, Bangladesh; 3 Department of Applied Statistics, Faculty of Economics and Administration, University of Malaya, Kuala Lumpur, 50603, Malaysia; 4 Department of Public Health Medicine, School of Public Health, Bielefeld University, Postfach 100131, Bielefeld, D-33501, Germany

**Keywords:** Developing country, Global youth tobacco Survey, Secondary school students, Tobacco consumption

## Abstract

**Background:**

Tobacco consumption (TC) among youths poses significant public health problem in developing countries. This study utilized the data of Global Youth Tobacco Survey (GYTS), 2007 to examine and compare youth TC behavior in Bangladesh, Nepal and Sri Lanka.

**Methods:**

The GYTS covered a total of 2,242 Bangladeshi, 1,444 Nepalese and 1,377 Sri-Lankan youths aged 13–15 years. They represented response rates of 88.9%, 94.6%, and 85.0% for the three countries, respectively. Socioeconomic, environmental, motivating, and programmatic predictors of TC were examined using cross tabulations and logistic regressions.

**Results:**

Prevalence of TC was 6.9% (9.1% in males, 5.1% in females) in Bangladesh, 9.4% (13.2% in males, 5.3% in females) in Nepal and 9.1% (12.4% in males, 5.8% in females) in Sri Lanka. The average tobacco initiation age was 9.6, 10.24 and 8.61 years, respectively. Cross tabulations showed that gender, smoking among parents and friends, exposure to smoking at home and public places, availability of free tobacco were significantly (P < 0.001) associated with TC in all three countries. The multivariable analysis [odds ratio (95% confidence interval)] indicated that the common significant predictors for TC in the three countries were TC among friends [1.9 (1.30-2.89) for Bangladesh, 4.10 (2.64-6.38) for Nepal, 2.34 (1.36-4.02) for Sri Lanka], exposure to smoking at home [1.7 (1.02-2.81) for Bangladesh, 1.81 (1.08-2.79) for Nepal, 3.96 (1.82-8.62) for Sri Lanka], exposure to smoking at other places [2.67 (1.59-4.47) for Bangladesh, 5.22 (2.76-9.85) for Nepal, 1.76 (1.05-2.88) for Sri Lanka], and the teaching of smoking hazards in schools [0.56 (0.38-0.84) for Bangladesh, 0.60 (0.41-0.89) for Nepal, 0.58 (0.35-0.94) for Sri Lanka].

**Conclusions:**

An understanding of the influencing factors of youth TC provides helpful insights for the formulation of tobacco control policies in the South-Asian region.

## Background

Annually, tobacco consumption (TC) kills more than 5 million people worldwide and the number is projected to increase to 8 million by 2030. More than 80% of those deaths will be in low- and middle-income countries [1,2]. In South Asia, approximately 1.2 million people die every year from TC [1]. The prevalence of TC and the associated consequences are declining rapidly in developed countries. However, TC of any form and the resulting death rates are still high in developing countries [1]. The gap in death rates due to TC between developing and developed countries is expected to increase over the next several decades [3]. The increase in youth TC, not only in percentages but also in numbers, in developing countries [1] widens this gap further. The prevalence of youth TC in developing countries varies by country and gender. However, the males are more likely to consume tobacco than females [4,5] in general.

Notable theories such as the theory of triadic influence, social learning theory, social identity theory, primary socialization theory, social network theory, and theory of social development have been used to explain TC behavior among the youths and possible predictors of such behavior [6,7]. Besides, some studies reported socio-demographic and environmental conditions, parental and peer influence, as well as motivational and programmatic dispositions as likely reasons of youth TC [8,9]. Family history of smoking and peer influence has substantially increased the likelihood of smoking initiation among youth at an earlier age compared to those living in a non-smoking environment [8,10]. Youths exposed to smoking at home generally perceived smoking as a social norm and have the tendency to follow this risky behavior [5]. Similarly, exposure to smoking at schools and other places also influenced the smoking behavior of youth, as they feel it is socially acceptable to smoke when the phenomenon is common occurrence of daily life [11].

Tobacco-related consequences on the youths are different from those on adults. Youths are more vulnerable to develop strong addictive behavior that has a high likelihood to result in long-term tobacco use. If addicted from a young age of tobacco initiation, youths can continue to smoke for long periods of time. They are also replacements for smokers who quit or die, and therefore remain the targets of tobacco industries [1].

This study aims to compare youth tobacco consumption in three selected countries of South Asia, namely, Bangladesh, Nepal and Sri Lanka. Our study is important for a few reasons. First, studies concerning youth TC in developing countries and factors that determine smoking are comparatively scarce compared to the literature on developed countries. Second, studies focusing on cross-country comparisons in South Asia are very limited and hence little is known about TC behavior in this region, especially that among the youths. Third, preventing youth from initiating TC is a major goal in public health particularly in developing countries, and therefore a better understanding of youth TC will serve as a guide for policy formulation. Fourth, despite established country-specific tobacco control laws and policies, rising prevalence of youth TC and nicotine dependence is still a major concern in South Asia [1]. Fifth, the tobacco industries are shifting their business focus from developed to developing countries and targeting the youths especially girls, whose TC prevalence is lower than their male counterparts [1]. For the above reasons, the findings of this study based on nationally representative data collected with common methodology across the three countries shall form useful inputs for developing effective and dynamic strategies for TC control.

## Methods

### The data

This study used nationally representative data on youths from Bangladesh, Nepal and Sri Lanka collected through the Global Youth Tobacco Survey (GYTS 2007), which was designed and conducted by the World Health Organization (WHO) and Centers for Disease Control (CDC). The detailed methodology and data collection procedure were given in the country specific report of GYTS 2007 [12-14]. Briefly, GYTS is a school-based survey that employed a two-stage cluster sampling design. The sampling frame included all the secondary-level schools in Bangladesh, Nepal and Sri Lanka containing classes of grades 7 to 10. In the first sampling stage, schools were selected with probabilities that were proportional to the number of students enrolled in the specified grades. At the second stage, classes within these schools were randomly selected. All students in the selected classes who attended school on the days of survey were eligible to participate. For Bangladesh and Sri Lanka, the response rates at the school level were 100% whereas for Nepal the rate was 98%. The response rates of students varied across the countries with 88.9% in Bangladesh, 96.6% in Nepal and 85.0% in Sri Lanka. Thus, the overall response rates (the response rates of the schools multiplied by those of the students) were 88.9% in Bangladesh, 94.6% in Nepal and 85.0% in Sri Lanka. Weighted data by schools, classes and students were used to avoid non-response bias. The dataset included 2242, 1444 and 1377 youths aged 13–15 years from Bangladesh, Nepal and Sri Lanka respectively.

### Questionnaire

The GYTS questionnaires were self-administered in classrooms. Anonymity of schools, classes, and students was maintained throughout the GYTS process. From a large set of variables (responses to the core questions of GYTS as well as country-specific questions), we selected the relevant variables where information were consistently collected across the three countries for this study. The response variable is current tobacco use, defined as below:

Having used cigarettes, *bidis*^a^ or other smoked tobacco products,^b^ or smokeless tobacco^c^ at least once during the past 30 days before the survey. The variable is coded “yes” or “1” if the respondent has used any of these tobacco products at least once and “no” or “0” otherwise.

The details of the variables used in this study and how they were coded for analysis are presented in Table 1.

**Table 1:** Variables included in the study and their coding for analysis

**Variable**	**Question asked in the survey**	**Coding for analysis**
	**Response variable**	
	**(a) Smoked tobacco**	
Variable ID: CR3 BDR15 NPR12	During the past 30 days, on how many days did you smoke **cigarettes/bidis**? Options included: 1 = 0 days, 2 = 1 or 2 days, 3 = 3 to 5 days, 4 = 6 to 9 days, 5 = 10 to 19 days, 6 = 20 to 29 days, 7 = All 30 days	0 = Not using cigarettes (option 1) 1 = Any use (option 2 to 7)
Variable ID: NPR17 CR8	During the past 30 days, did you use any form of smoked tobacco products other than **cigarettes** and ***bidis*** such as cigars, water pipe (*hukkah*), cigarillos, little cigars, pipe etc.? Options included: 1 = yes, 2 = no	0 = Not using other smoked tobacco products (option 2) 1 = Any use (option 1)
	**(b) Smokeless tobacco**	
Variable ID: BDR23 NPR20	(A) During the past 30 days, on how many days did you use smokeless tobacco (chewing or applying or snuff) such as *surti*, *khaini*, *panmasala*, *gutka*, *parag*, *gul* etc.? Options included: 1 = 0 days, 2 = 1 or 2 days, 3 = 3 to 5 days, 4 = 6 to 9 days, 5 = 10 to 19 days, 6 = 20 to 29 days, 7 = All 30 days	0 = Not using any smokeless products (option 1) 1 = Any use (option 2 to 7)
Variable ID: CR9	(B) During the past 30 days, did you use any form of smokeless tobacco products (e.g. chewing tobacco, snuff, dip)? Options included: 1 = yes, 2 = no	0 = Not using any smokeless tobacco products (option 2) 1 = Any use (option 1)
Variable Name	Question asked in the survey	Coding for analysis
**Independent variables**		
Age in years CR52	How old are you? Options included: 11 to 17 years	1 = youths **(13 to 15 years of age);** 0 = others
Gender: CR53	What is your sex?	1 = male, 2 = female
Education gradeBDR73, NPR75, LKR54	In what grade are you?	Seventh, Eighth, Ninth, Tenth
Parental tobacco use CR12, BDR25, BDR26	Do your parents smoke cigarettes/*bidis* or use smokeless tobacco? 1 = none, 2 = both, 3 = father only, 4 = mother only, 5 = I don’t know	0 = no (option 1 & 5) 1 = yes (option 2 to 4)
Friends tobacco use CR25	Do any of your closest friends smoke? 1 = none of them, 2 = some of them, 3 = most of them, 4 = all of them	0 = no (option 1) 1 = yes (option 2 to 4)
Smoking at home CR30	During the past 7 days, on how many days have people smoked in your home, in your presence? Options included:	0 = no (option 1) 1 = 1-4 days (option 2 & 3) 3 = 5-7 days (option 4 & 5)
Smoking at other places than home CR31	During the past 7 days, on how many days have people smoked in your presence, in places other than in your home? 1 = 0, 2 = 1 to 2, 3 = 3 to 4, 4 = 5 to 6, 5 = 7 days	0 = no (option 1) 1 = 1-4 days (option 2 & 3) 3 = 5-7 days (option 4 & 5)
Offer free tobacco products by sales men: CR47	Has a cigarette company representative ever offered you a free cigarette? Options included: 1 = yes, 2 = no	0 = no (option 2) 1 = yes (option 1)
Advertisement seen in hoarding, bus-stop, station CR44	During the past 30 days, how many advertisements for cigarettes have you seen on hoardings, buses, bus-stops, trains, railway platforms, shops or as writings on walls? Options included: 1 = a lot, 2 = a few, 3 = none	0 = none (option 3) 1 = a few (option 2) 2 = a lot (option 1)
Taught in class about danger of smoking: CR48	During this school year, were you taught in any of your classes about the dangers of smoking? Options included: 1 = yes, 2 = no, 3 = not sure	0 = no (option 2 and 3) 1 = yes (option 1)
Discussed smoking and health as part of a lesson BDR70, CR51	How long ago did last discuss smoking and health as part of a lesson? 1 = never, 2 = this year/term, 3 = last year/term, 4 = 2 years/terms ago, 5 = 3 years/terms ago, 6 = more than 3 years/terms ago	0 = never (option 1) 1 = during this survey year (option 2); 2 = before year of survey (option 3 to 6)

CR indicates the core questions that were asked for all countries in GYTS. Country specific questions were labeled as BDR for Bangladesh, NPR for Nepal and LKR for Sri Lanka.

To examine the influencing factors, we selected eleven independent variables categorized into four different groups. The selection was based on the literature [4,15-20] and theories [6,7] on youth TC. The four categories of variables are:

(i) background factors – age, sex and education grade, (ii) environmental factors – tobacco use behavior of parents and friends, and whether they smoked at home or public places in the presence of respondent, (iii) motivating factors – free tobacco products from the vendors, advertisements and promotions in mass media and other places (iv) programmatic factors – whether taught in class about the danger of smoking and discussed smoking and health as part of a lesson.

### Statistical analysis

First, we provided the descriptive information about the sample of study based on frequency runs. Second, bivariate cross tabulations of the response variable on the independent variables were generated. The chi-square tests of association between the response and independent variables were conducted and the P-values of the test statistics for testing the null hypothesis of no association were reported [21]. Finally, we performed the multivariable logistic regression analysis [22]. The logistic regression model is given by:

$$\text{Pr}\left(Y_{i}=1\right)=\frac{\text{exp}\left(X_{i}\beta \right)}{1\;+\;\text{exp}\left(X_{i}\beta \right)}$$

where Y_i_ is a binary variable that takes a value of ‘1’ if the respondent is a tobacco user and ‘0’ otherwise, X_i_ is a vector of independent variables and *β* is a vector of unknown parameters. Tobacca users refer to those who used any form of tobacco at least once in the past 30 days before the survey. The estimated form of the logistic transformation can be expressed as

$$\begin{array}{l} \text{In}\left[\frac{P_{i}}{{1-P}_{i}}\right]{=\beta }_{o}{+\beta }_{1}X_{1}{+\beta }_{2}X_{2}{+\beta }_{3}X_{3}{+\beta }_{4}X_{4}{+\beta }_{5}X_{5}\\ \;{+\beta }_{6}X_{6}{+\beta }_{7}X_{7}{+\beta }_{8}X_{8}{+\beta }_{9}X_{9}{+\beta }_{10}X_{10}{+\beta }_{11}X_{11} \end{array}$$

The odds ratio (OR) in favor of tobacco consumption together with its 95% confidence interval (CI) were computed for X_1_, *X*_2_, … and X_11_ to measure how many times the group of interest is more likely to be a tobacco consumers compared to the reference group. The data were analyzed using SPSS (version 18; SPSS Inc, Chicago, IL).

## Results

Background information related to the respondents is given in Table 2. The proportion of the respondents in each age category ranged from 21-36% for Bangladesh, 26-37% in Nepal and 23-39% in Sri Lanka. Almost half of the respondents are females for Sri Lanka, while the proportion was 54% for Nepal and 59% for Bangladesh. Some 55% of the Bangladeshi and 66% of the Nepalese respondents were in junior classes of grade seven and eight. For Sri Lanka, a higher proportion of the respondents (70.2%) were in grade nine and ten.

**Table 2:** Background information of respondents, exposure to tobacco products and tobacco consumption for selected South-Asian countries, GYTS 2007

**Characteristics/Tobacco use**	**Bangladesh (2242)**		**Nepal (1444)**		**Sri Lanka (1377)**	
	**No.**	**%**	**No.**	**%**	**No.**	**%**
**Age in years**						
13	802	35.8	374	25.9	522	37.9
14	961	42.9	535	37.1	539	39.1
15	479	21.4	534	37.0	316	22.9
**Gender**						
Female	1301	59.2	776	53.8	691	50.2
Male	897	40.8	667	46.2	685	49.8
**Educational grade**						
Seventh	584	26.1	461	32.0	-	-
Eighth	644	28.7	494	34.3	395	29.8
Ninth	581	25.9	331	23.0	520	39.1
Tenth	433	19.3	156	10.8	414	31.1
**Parental tobacco use**						
No	1028	45.9	738	51.2	954	70.1
Yes	1210	54.1	705	48.8	406	29.9
**Friends’ tobacco use**						
No	1711	76.3	1041	72.7	1144	84.4
Yes	531	23.7	392	27.3	211	15.6
**Smoking at home in last 7 days in presence of youths**						
No	1463	65.3	927	64.7	874	64.6
1-4 days	385	17.2	273	19.0	380	28.1
5-7 days	392	17.5	234	16.3	100	7.4
**Smoking at other places in last 7 days in presence of youths**						
No	1286	57.8	760	52.7	464	34.1
1-4 days	494	22.2	503	34.9	533	39.2
5-7 days	444	20.0	180	12.5	362	26.6
**Offering free tobacco products by sales men**						
No	1938	89.0	1180	83.0	1291	97.0
Yes	240	11.0	242	17.0	40	3.0
**Advertisement seen in hoarding, bus-stop, rail stations**						
None	593	26.5	220	15.3	438	32.6
A few	760	34.1	685	47.8	678	50.5
A lot	880	39.4	529	36.9	227	16.9
**Taught in class about danger of smoking**						
No	1021	45.8	486	34.2	358	27.2
Yes	1211	54.2	937	65.8	958	72.8
**Discussed smoking & health as part of a lesson**						
Never	1444	64.7	522	36.2	203	15.3
During the survey year	583	26.1	397	27.6	268	20.2
Preceding years of survey	206	9.2	520	36.2	858	64.6
Tobacco use prevalence and average daily consumption in last 30 days						
Male & female	156	6.9	136	9.4	125	9.1
Male	85	9.1	101	13.2	85	12.4
Female	71	5.1	35	5.3	40	5.8
Average number of cigarettes smoke per day	0.93		1.32		3.22	
Average number of *bidis* smoke per day	1.62		0.75		-	
Average age (years) of tobacco initiation	9.6		10.24		8.61	

^**¶**^The total does not always add up due to missing values.

Table 2 also provides information on environmental tobacco smoke (ETS), exposure to tobacco products and tobacco use behavior. At least one of the parents of about 54% of the respondents from Bangladesh was a smoker. The proportion was slightly lower for Nepal (49%) and the lowest in Sri Lanka (30%). The incidence of TC among their friends was about 24% in Bangladesh and 27% in Nepal, but lowest in Sri Lanka (16%). About 35% of the youths in all the three countries were exposed to smoking by family members, friends or visitors in their home within seven days preceding the survey. Some 42.2% of the respondents in Bangladesh, 47.4% in Nepal and 65.8% in Sri Lanka witnessed someone smoking at public places.

Nepal had the highest proportion (17%) of youths who received free tobacco products from salesmen. In contrast, this proportion was the lowest in Sri Lanka (3%). About 33% of the youths from Sri Lanka compared to 15.3% from Nepal were never exposed to any advertisements in hoarding, bus-stop and railway stations. Majority of the Sri Lankan (73%) and Nepalese (66%) youths were taught in schools about the danger of smoking, but such lessons were less common to the youths from Bangladesh. More than 60% of the Bangladeshi youths reported that issues on smoking and health were not discussed as part of school curricular. In contrast, only 36.2% of the Nepalese and 15.3% of the Sri Lankan respondents had reported the same.

The problem of youth tobacco use seemed more serious in Nepal and Sri Lanka compared to Bangladesh. This risky behavior was also more widespread among the males in all the three countries. Its prevalence rate was 6.9% (9.1% in males, 5.1% in females) among the youths in Bangladesh, 9.4% (13.2% in males, 5.3% in females) in Nepal and 9.1% (12.4% in males, 5.8% in females) in Sri Lanka. The average age of smoking initiation was 9.6, 10.24 and 8.61 years for the three countries respectively.

**Figure 1:**
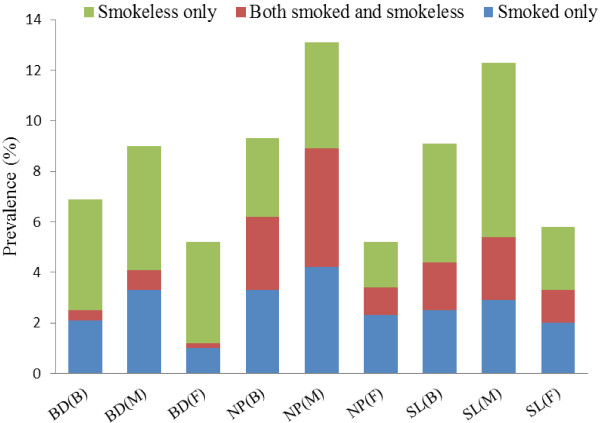
**Types of current tobacco use among youths aged 13 to 15 years in selected South Asian countries, global youth tobacco survey, 2007.** Notes: BD, NP and SL represent Bangladesh, Nepal and Sri Lanka. B represents both gender, M for male and F for female.

TC included smoked and smokeless tobacco products. Figure 1 shows the types of tobacco consumed among the youths by gender. It is clear that in Bangladesh and Sri Lanka, the use of smokeless tobacco constituted a major proportion of total TC. In Bangladesh, smokeless tobacco use was slightly higher among the males (4.9%) than the females (4%). In Sri Lanka, the males consumed more than double (6.9%) of smokeless tobacco products consumed by the females (2.5%). In Sri Lanka, dual users (both smoked and smokeless tobacco products) contributed significantly to total TC, and there were more male than female users in this category. However, in Nepal, TC had an almost equal distribution (2.9 to 3.3%) of the three categories of tobacco products. The difference between proportions of male and female consumers was highest for Nepal for all three types of tobacco products compared to the other two countries. The proportion of male consumers was more than double (triple in the case of dual users) of that of their female counterparts.

### Factors associated with tobacco consumption

TC in Bangladesh, Nepal and Sri Lanka was significantly (P < 0.001) influenced by gender, smoking history of parents and friends, exposure to smoking at home and public places, and availability of free tobacco products (Table 3). Respondents with parents or friends who were tobacco consumers and those exposed to smoking at home or public places had a higher tendency to smoke. The smoking prevalence rate was also higher among those who were offered free tobacco products. In at least one of the three countries, youth TC was also associated with other factors such as exposure to advertisements on tobacco products, and whether there were discussions of smoking hazards in school curricula. The Bangladeshi and Nepalese youths who were exposed to advertisements on tobacco products had a higher tendency to become smokers. Discussion of the danger of smoking in school curricula had helped to deter smoking, at least in Bangladesh and Sri Lanka.

**Table 3:** Factors associated with tobacco use among youths from selected South-Asian countries, GYTS 2007

**Factors**	**Bangladesh**		**Nepal**		**Sri Lanka**	
	**Tobacco**	**Chi-square**	**Tobacco**	**Chi-square**	**Tobacco**	**Chi-square**
	**Use (%)**	**(P-values)**	**Use (%)**	**(P-values)**	**Use (%)**	**(P-values)**
**Age in years**						
13	4.7		6.0		8.8	
14	6.7	22.28	8.3	14.49	8.7	3.66
15	11.6	(< 0.001)	13.2	(0.001)	12.4	(0.160)
**Gender**						
Female	5.1	13.59	5.3	25.6	5.8	16.87
Male	9.1	(< 0.001)	13.2	(< 0.001)	12.4	(< 0.001)
**Educational grade**						
Seventh	4.5		9.3		-	
Eighth	6.1		8.2		8.8	
Ninth	10.1	14.75	11.8	3.10	9.7	0.312
Tenth	7.7	(0.001)	9.8	(0.38)	8.8	(0.86)
**Parental tobacco use**						
No	4.6	15.93	7.3	8.2	8.2	4.27
Yes	8.9	(< 0.001)	11.7	(0.001)	11.9	(0.001)
**Friends’ tobacco use**						
No	4.5	70.34	4.1	124.32	6.6	58.76
Yes	15.2	(< 0.001)	23.5	(< 0.001)	24.2	(< 0.001)
**Smoking at home in last 7 days in presence of youths**						
No	4.4		5.6		6.3	
1-4 days	12.1	41.18	19.3	51.27	13.2	41.71
5-7 days	11.3	(< 0.001)	13.7	(< 0.001)	25.3	(< 0.001)
**Smoking at other places in last 7 days in presence of youths**						
No	3.9		3.6		6.4	
1-4 days	9.0	55.69	14.7	67.9	9.4	11.13
5-7 days	14.1	(< 0.001)	19.7	(< 0.001)	13.5	(.001)
**Offering free tobacco products by sales men**						
No	5.6	53.45	9.1	3.50	8.4	31.0
Yes	18.5	(< 0.001)	14.5	(< 0.001)	35.1	(< 0.001)
**Advertisement seen in hoarding, bus-stop, rail stations**						
None	4.9		7.9		8.3	
A few	5.1	18.3	10.0	.84	7.7	13.19
A lot	9.7	(< 0.001)	9.3	(0.656)	15.8	(0.001)
**Taught in class about danger of smoking**						
No	8.2	5.14	10.7	1.13	12.0	3.40
Yes	5.8	(0.023)	8.9	(0.28)	8.5	(0.05)
**Discussed smoking & health as part of a lesson**						
Never	5.6		11.4		10.7	
During the survey year	7.5	21.73	8.9	3.45	11.4	1.90
Preceding years of survey	14.5	(< 0.001)	8.2	(0.178)	8.8	(0.387)

### Factors influencing tobacco consumption behaviors

The results of the multivariable logistic regression analysis are given in Table 4. According to the results, the likelihood of TC was about 1.5 (95% CI of 0.90-2.64) times higher among youths aged 15 years in Bangladesh compared to those who were 13 years old. The corresponding odds ratios were 2.3 (95% CI of 1.20-4.37) for Nepal and 2.8 (95% CI of 1.05-7.30) for Sri Lanka. Significantly higher likelihood of TC was found among the males (OR = 1.31 for Bangladesh, OR = 1.77 for Nepal and OR = 2.77 for Sri Lanka). Parental TC did not have any significant effect on the tobacco use behavior of the youths. However, friends’ TC increased the likelihood of smoking among the respondents by at least two times (OR = 1.9 with 95% CI of 1.30-2.89 for Bangladesh, OR = 4.1 with 95% CI of 2.64-6.38 for Nepal, and OR = 2.3 with 95% CI of 1.36-4.02 for Sri Lanka). The impact of influence from friends on smoking is the highest in Nepal.

**Table 4:** The odds ratio and 95% confidence interval for factors influencing tobacco use among youths from selected South-Asian countries, GYTS 2007

**Factors**	**Bangladesh**		**Nepal**		**Sri Lanka**	
	**OR**	**95% C.I**	**OR**	**95% C.I**	**OR**	**95% C.I**
**Age in years**						
13	-	-	-	-	-	-
14	1.13	(0.69-1.86)	1.47	(0.78-2.76)	0.96	(0.45-2.04)
15	1.54	(0.90-2.64)	2.29**	(1.20-4.37)	2.77**	(1.05-7.31)
**Gender**						
Female	-	-	-	-	-	-
Male	1.31	(0.89-1.95)	1.77**	(1.10-2.87)	2.12***	(1.29-3.50)
**Educational grade**						
Seventh	-	-	-	-	-	-
Eighth	1.02	(0.57-1.83)	0.63	(0.36-1.10)	-	-
Ninth	1.40	(0.77-2.54)	1.35	(0.76-2.39)	1.40	(0.65-3.02)
Tenth	1.39	(0.76-2.53)	1.25	(0.65-2.32)	1.29	(0.66-2.38)
**Parental tobacco use**						
No	-	-	-	-	-	-
Yes	1.04	(0.68-1.60)	1.10	(0.69-1.88)	1.05	(0.69-1.61)
**Friends’ tobacco use**						
No	-	-	-	-	-	-
Yes	1.94***	(1.30-2.89)	4.10***	(2.64-6.38)	2.34***	(1.36-4.02)
**Smoking at home in last 7 days in presence of youths**						
No	-	-	-	-	-	-
1-4 days	1.80**	(1.09-2.96)	1.98**	(1.17-3.35)	2.25***	(1.31-3.88)
5-7 days	1.70**	(1.02-2.81)	1.81**	(1.08-2.79)	3.96***	(1.82-8.62)
**Smoking at other places in last 7 days in presence of youths**						
No	-	-	-	-	-	-
1-4 days	1.67*	(0.98-2.85)	3.36***	(1.93-5.87)	1.44	(0.80-2.57)
5-7 days	2.67***	(1.59-4.47)	5.22***	(2.76-9.85)	1.76*	(1.05-2.88)
**Offering free tobacco products by sales men**						
No	-	-	-	-	-	-
Yes	3.08***	(1.97-4.83)	1.31	(0.71-2.42)	2.57*	(0.93-7.09)
**Advertisement seen in hoarding, bus-stop, rail stations**						
None	-	-	-	-	-	-
A few	0.90	(0.51-1.59)	0.91	(0.49-1.69)	0.89	(0.51-1.53)
A lot	1.46	(0.87-2.45)	0.94	(0.50-1.82)	1.61	(0.85-3.06)
**Taught in class about danger of smoking**						
No	-	-	-	-	-	-
Yes	0.56***	(0.38-0.84)	0.60*	(0.41-0.89)	0.58**	(0.35-0.94)
**Discussed smoking & health as part of a lesson**						
Never	-	-	-	-	-	-
During the survey year	0.79	(0.47-1.56)	0.45***	(0.25-0.78)	0.66*	(0.41-0.99)
Preceding years of survey	0.67**	(0.43-0.92)	0.45***	(0.27-0.73)	0.67*	(0.42-1.02)

*P < 0.05, **P < 0.01, ***P < 0.001 (P-values); odds ratio (OR), confidence interval (C.I).

ETS from smoke at home and public places also appeared as a significant risk factor for TC. For instance, if the youths were exposed to others smoking at home in the preceding seven days before the survey, the likelihood of them involved in TC was at least two times higher than the youths who did not have a similar exposure. This imitation behavior was more common among the Sri Lankan youths compared to the other two countries. On the contrary, although secondhand smoke exposure from public places increased the likelihood of TC in all three countries, its impact was higher on the youths in Bangladesh and Nepal.

The provision of free tobacco products by vendors had influenced TC behavior among the youths. For instance, the likelihood of TC was 3 times higher among the Bangladeshi youths and 2.6 times higher among the Sri Lankan youths who had received free tobacco products from the vendors. Teaching in class about the danger of smoking had reduced the likelihood of smoking to almost half for all the three countries. Similarly, discussions on smoking and health in school curriculum had also reduced TC tendency.

## Discussion

The overall prevalence of youth TC in the three selected countries was below 10% with significantly higher rates in the males. A comparison can be made with the prevalence rates of other countries reported in [1]. The rates reported in this study were lower than those in other South-Asian countries like Bhutan (18.3% in males, 6.3% in females) and India (16.8% in males, 9.4% in females), comparable to Pakistan (12.4% in males, 7.5% in females), but higher than Maldives (8.5% in males, 3.4% in females). The three selected countries also had higher youth TC rates than some neighboring countries such as China (7.1% in males, 3% in females), but lower than Myanmar (22.5% in males, 8.2% in females) and Thailand (21.7% in males, 8.4% in females). Consistent with the findings on other South-Asian and neighboring countries, this study showed significantly higher TC prevalence among the males. The overall TC among female youths in the countries selected for this study was between 5-6%, but self-reporting in the survey and conservative social structure in these countries may lead to under-reporting of the actual situation. It should also be noted that the male–female differentials in TC prevalence rate in the three selected countries were much lower than those of some countries reported in [1], including Bhutan, India, Myanmar and Thailand. It is expected that in near future TC among female youths will be high in this region [1]. This assumption about female TC may be due to the overall impact of globalization, urbanization, marketing efforts of the tobacco industry, and changing status of women from higher educational attainment and better employment opportunities [5,16]. Our findings are supported by the theory of triadic influence [23] and other studies [5,17].

In contrast with other studies [5,10], parental TC in the selected countries did not show any effect on their children’s tobacco usage. However, in accordance with some studies [4,8,24-26], the likelihood of TC increased significantly among youths who had witnessed others smoking at home and public places. This ETS exposure not only created health hazards but also influenced them to initiate TC. Such exposure impacted more on the Nepalese and Sri Lankan youths compared to their counterparts from Bangladesh.

Having seen friends using tobacco products increased the likelihood of TC among youths [5,7,15]. In line with some empirical evidence in the literature and theories, this study showed that the risk of TC were 2 to 4 times higher due to peer influence. This could be due to imitation, peer pressure or group characteristics. Peer influence had the largest impact on TC in Nepal than in Bangladesh and Sri Lanka.

Easy or sometimes free access to tobacco products and lack of restrictions on sales to minors increased the possibility of TC among youths [4,27,28]. Law enforcement, whether in or outside school compound, and tobacco control measures are essential [1,12,14]. Of the three countries, strict enforcement of law was most evident in Sri Lanka. This country had the lowest percentage of youths that were offered free samples by tobacco vendors, and those who had seen tobacco advertisements on hoardings, bus stops and rail stations. In contrast, Nepal had the highest incidence of tobacco sample handouts and tobacco advertisements. On tobacco control measures, Sri Lanka also appeared to have done the most through education of TC hazards. Bangladesh, on the other hand, had lagged behind the two other countries in educating the youths on the adverse effects of tobacco use in schools.

Consistent with other findings, this study showed that free tobacco products from vendors had significantly influenced TC behavior of the Bangladeshi and Sri Lankan youths. This may be related to other factors such as school environment [11,17], cultural norms [5,17], socio-economic reasons [8,16] and psychological factors [23,27]. Although free tobacco products were relatively easier to obtain in Nepal compared to the two other countries, the Nepalese youths were not as easily influenced, perhaps due to different level of knowledge, attitudes and awareness. For the same reasons, incorporation of health issues and hazards of smoking in school lessons had a higher positive impact on the Nepalese youths. Knowledge about the danger of addictive tobacco behavior significantly reduced future TC [8,9]. This study found that class lessons on the danger of smoking and discussions of smoking and health as part of school lessons had reduced tobacco usage among the youths in all three countries.

### Data limitations and future direction

The GYTS was based on self-reporting and therefore, is subject to recall bias and deliberate misreporting. Even though anonymity was emphasized by the WHO officials and efforts were made to assure confidentiality, respondents may have under- or over-reported their actual smoking status given that TC is not a widely acceptable social norm in the South-Asian region. In addition, GYTS is a school based survey that reflects the opinion of students about TC, which may not represent the views of all the youths, especially of those who are not schooling. In order to overcome such difficulties and to ensure data reliability, collected data could be verified by biomarkers using cotinine or exhaled carbon monoxide assessments. These techniques will be useful for obtaining more accurate responses. The variables utilized for statistical analysis were limited to those available in the dataset. However, there may be other important variables that were not considered. A qualitative study is suggested to supplement the understanding of the determinants of the tobacco use behavior among youths. Since the data is cross-sectional, causal relationships cannot be inferred.

## Conclusion

Although Bangladesh, Nepal and Sri Lanka have country-specific tobacco control laws and policies, TC among the youths is common and the magnitude of the problem is increasing. We identified several factors that influenced youth TC behavior and suggested some guidelines for policy purpose. First, the impact of peer influence could be reduced through close monitoring by parents and schools. Parents and teachers have a role to play to prevent the youths from subjecting to peer pressure. Youth smoking should be curbed at an early stage to prevent addiction and further spread of the problem. Second, elimination of passive smoking (e.g., posing more barriers at home and other public places to prevent smoking in the presence of youths), punishments for tobacco vendors who violate existing laws or offer free tobacco products to minors, and restrictions of advertisements in hoardings, bus-stops and rail stations should be geared up to reduce TC and increase tobacco cessation among the youths in this region. Third, school curricula should be revised to meet country-specific needs to emphasize tobacco prevention and to increase awareness of its consequences among the youths. Besides, no smoking should be allowed in school compounds as students can imitate the smoking behavior of teachers or other staff. This should be monitored by school disciplinary committees on a regular basis. Attention should be on the boys in policy formulation as they tend to be more vulnerable to smoking. However, the girls, particularly from developing countries, should not be neglected as they are increasingly being targeted by tobacco companies.

Given the above issues, parents, teachers, sports and media personalities, religious and community leaders, government agencies, and regional organizations should work together to develop new tobacco control programs, while ensuring full enforcement of existing laws. If the issue of TC is not addressed, the associated health and social problems will continue to spread and drain economic resources for dealing with the problems. Given that GYTS is a school based survey, the prevalence of TC among students has further economic and social implications. Any negative health outcomes due to smoking may directly affect the academic performance of the youths, causing absenteeism in schools and increasing dropout rates, all of which bear high social and economic costs. In addition, smoking creates additional strains on family budget especially for the poor, making it more difficult for them to break the poverty cycle. TC will indirectly increase government expenditure on health, education and programs to redress the social and economic problems associated with smoking. Therefore, comprehensive strategies along with preventive programs should be implemented effectively to help youths avoid smoking, quit such hazardous behavior, and prevent life-long addiction.

## Endnotes

^a^ Made of a small amount of crushed tobacco, hand-wrapped in dried *tendu* leaves, and tied with string. Despite their small size, *bidis* tend to deliver more tar and carbon monoxide than manufactured cigarettes because users must puff harder to keep them lit.

^b^ Such as cigars, water pipe (*hukkah*), cigarillos, little cigars, pipe etc.

^c^ Smokeless tobacco products such as *surti, khaini, panmasala, gutka, parag, gul* etc.

## Competing interests

The authors declare that they have no competing interests.

## Authors’ contributions

MAK and MMHK conceptualized the research topic and drafted the manuscript. MAK performed the data analysis in consultation with KLG and MMHK. KLG contributed to the writing process, interpretation of results and revised the article critically. MMHK also provided further inputs in the revision stage. MAK structured the manuscript, collected the references and finalized the paper for submission. All authors read and approved the final manuscript.

## Pre-publication history

The pre-publication history for this paper can be accessed here:

http://www.biomedcentral.com/1471-2458/13/379/prepub
